# Genetic diversity of thrips populations on *Allium* species around the world

**DOI:** 10.1371/journal.pone.0289984

**Published:** 2023-08-17

**Authors:** Bettina Porta, Ben Vosman, Richard G. F. Visser, Guillermo A. Galván, Olga E. Scholten

**Affiliations:** 1 Plant Breeding, Wageningen University and Research Centre, Wageningen, The Netherlands; 2 Departamento de Biología Vegetal, Facultad de Agronomía, Universidad de la República, Montevideo, Uruguay; 3 Departamento de Producción Vegetal, Centro Regional Sur (CRS), Facultad de Agronomía, Universidad de la República, Progreso, Canelones, Uruguay; Gomal University, PAKISTAN

## Abstract

Thrips are a serious pest in many crops. In onion cultivation, *Thrips tabaci* is the most important, but not the only thrips species causing damage. We investigated which thrips species affects onion and related species worldwide, how much genetic variation there is within *T*. *tabaci* populations, and how this evolves. Furthermore, we determined the reproductive mode and the correlation between the genetic and geographic distances. Thrips samples from infested onions or related species were obtained from 14 different locations worldwide. Species and haplotypes were determined through DNA barcoding with the mitochondrial Cytochrome Oxidase subunit I (COI) gene. *Thrips tabaci* was the most commonly observed species, but *Scirtothrips dorsalis*, *Thrips palmi*, *Frankliniella intonsa*, *Frankliniella occidentalis* and *Frankliniella tenuicornis* were also found, especially at the beginning of the growing seasons and depending on the location. The Nei’s genetic distance within *T*. *tabaci* was less than 5% and the haplotypes were clustered into two phylogenetic groups, each linked to a specific mode of reproduction, thelytokous or arrhenotokous. Thelytokous thrips were more common and more widely distributed than arrhenotokous thrips. A high percentage of heteroplasmy was detected in the arrhenotokous group. Heteroplasmic thrips were only found in populations where thelytokous and arrhenotokous were present in sympatry. Some *T*. *tabaci* haplotypes were present in high frequency at several sampled locations. No correlation was found between the genetic and geographic distances, which points to anthropic activities spreading thrips haplotypes throughout the world.

## Introduction

Thrips are small insects of around 1 mm long that belong to the order Thysanoptera. This order contains 6,200 species [1], all with fringed wings and asymmetrical mouthparts. Most species feed on plants by puncturing the cells and sucking up their content, while just a few species are predators of other insects or mites [2]. A small number of thrips species are beneficial as pollinators. Nevertheless, several thrips, especially the species that show high adaptability and a polyphagous nature, are serious pests in commercially important crops [2].

*Thrips tabaci* Lindeman (Thysanoptera: Thripidae), commonly known as onion thrips [3], is polyphagous and occurs worldwide. It infests a wide range of plant species including vegetables, fruits, and ornamentals [4]. *Thrips tabaci* is the major pest in onions worldwide [4]. It feeds directly on onion foliage, diminishing the photosynthetic capacity which can cause a more than 50% yield loss in commercial bulb production [5, 6]. *Thrips tabaci* has a high reproductive rate [4], shows cryptic behavior, is resistant to several insecticides [7, 8], and acts as a vector of plant pathogens [9, 10], including tospoviruses such as Iris Yellow Spot Virus (IYSV) which can devastate an onion crop in a matter of weeks [6, 11]. All these characteristics make it a high-impact pest that is difficult to control [12].

*Thrips tabaci* comprises three phylogenetic groups associated with different host plants or reproductive modes [13]. One group is mainly found on tobacco plants [14]. The other two groups are mainly associated with onion and leek and correspond to the major reproductive modes, arrhenotokous and thelytokous [14, 15]. The arrhenotokous and thelytokous lineages differ in ecological characteristics like host preference [16] and pyrethroid resistance [7].

In the case of thelytokous thrips, reproduction is asexual by parthenogenesis in which the embryos develop into female offspring without fertilization. Arrhenotokous thrips can reproduce both sexually and asexually, producing female offspring from fertilized eggs [17], and haploid males from unfertilized eggs [15]. In rare cases, deuterotokous thrips that develop from unfertilized eggs in either male or female offspring via parthenogenesis have been reported [18]. A recent study established that deuterotoky in *T*. *tabaci* corresponds to an irregular mode of reproduction of the arrhenotokous thrips on tobacco [19]. The most common modes of reproduction, thelytoky, and arrhenotoky, are mainly mutually exclusive [20], but crosses between individuals belonging to these reproductive modes have been reported [21]. Such crosses may give thrips the ability to adapt more rapidly to changing environments, cope with pesticide pressures, and successfully infect various plant species [22].

*Thrips tabaci* originates from the Mediterranean region where onion is the main host plant [23]. From there, it has spread all over the world, colonizing habitats from sea level up to 2000 m [3]. How *T*. *tabaci* was distributed over the world is unknown. In a study with Chinese *T*. *tabaci* populations, no correlation was observed between genetic and geographic distances [24]. This may point to the migration of individual thrips, possibly through different routes, followed by adaptation to the new environment. Molecular data indicated that the three different phylogenetic groups in *T*. *tabaci* are not evenly distributed around the world [16].

Genetic diversity has different hierarchical levels, between and within species. The within-species variation can be subdivided into intra and inter-population diversity. How the variation is distributed over populations is highly influenced by reproductive mode, gene flow, and selection [25]. Genetic diversity within populations is determined by the presence of different genotypes, their relative frequencies, and the genetic distance between them.

Different genotypes of thrips can be discriminated by sequencing (part of) the Cytochrome Oxidase subunit I (COI) gene [13, 17, 24, 26]. The COI gene is part of the maternally inherited mitochondrial DNA and is commonly used for DNA barcoding of animals, including insects, to identify species. The mitochondrial genome is haploid and the genotypes distinguished are called haplotypes. Previous studies on genetic diversity in the *Thrips* genus showed that the COI gene is ideal for the simultaneous detection of intra- and interspecific genetic diversity, as the range of diversity within and between species does not overlap; between thrips species about 20% of genetic diversity is detected in the COI gene sequence, while within *T*. *tabaci* the genetic diversity is below 5% [26].

The COI gene can also be used to distinguish the different *T*. *tabaci* reproductive modes (or lineages), which are impossible to discriminate morphologically, either by using the COI sequence directly or by using reproductive-mode specific primers [15]. The mode-specific primers produce fragments of a specific length for each reproductive mode. In *T*. *tabaci* the presence of heteroplasmic individuals has been reported. Heteroplasmic thrips contain mitochondria with copies of the COI gene from different lineages [27], and in these cases the reproductive mode cannot be determined conclusively using specific primers; however, the reproductive mode may be deducted from their position in the phylogenetic tree or determined by a progeny test from virgin females [13].

Although *T*. *tabaci* is the most important pest in onion [5], other thrips species have been found on onion as well [28]. It is important to know which thrips species affect onion, their genetic diversity and distribution around the world, as well as the seasonal dynamics in the populations and the reproductive mode to develop improved control strategies to be implemented in future Integrated Pest Management programs [29]. This research aimed to 1) identify thrips species in fields with onions or onion-related species at 14 locations around the world; 2) determine genetic variation and mode of reproduction within *T*. *tabaci*, and relate these with the haplotypes reported in the NCBI sequence database; 3) compare the genetic variation within and between sampled thrips per location; 4) analyze the geographical distribution of the *T*. *tabaci* haplotypes as well as the correlation between the genetic and geographic distances of populations; and 5) analyze the seasonal variation in the composition of thrips populations at three locations.

## Materials and methods

### Thrips samples

Thrips samples were collected from onion or onion-related species present in experimental fields aimed to identify thrips resistance at 14 locations around the world (Table 1). Adult thrips or larvae (n>60) were randomly collected from different plants and stored in an Eppendorf tube containing 95% ethanol. Thrips were collected once (referred to as location) or at two or three different times (referred to as location-time) during the season (Table 1). Thrips samples were stored at room temperature prior to DNA extraction.

**Table 1:** Thrips sampling sites and passport data.

Continent	Country	Location	Latitude (dec)	Longitude (dec)	Host plant	Sowing and/or Planting date	Previous crop	Surrounding crop	Sampling date
**EUROPE**	**The Netherlands**	De Kwakel	52.2391	4.7940	*Allium* spp.			Grasses	26.07.2019
		Krabbendam	52.7318	4.7037	*Allium* spp.	29.03–22.05.2019			19.08.2019
		Wageningen	51.9869	5.6644	Rearing		---------	---------	11.03.2020
	**Spain**	Pulpi	37.3978	-1.7333	*Allium* spp.	10.4–19.6.2019			11.2019
									01.2020
	**France**	Eyragues	43.8510	4.8296	*Allium cepa* L.				09.07.2020
**ASIA**	**Israel**	Berurim	31.7712	34.7700	*Allium* spp.				16.03.2020
	**Japan**	Konan	34.9000	136.1000	*Allium* spp.			Bunching *Allium cepa* L. and *Allium cepa* L.	08. 08. 2019
									12. 09. 2019
									26. 09. 2019
	**Thailand**	Sansai	18.9749	98.9879	*Allium* spp.		Pepper	Bananas, bamboo, marigolds and weeds	21.01.2020
									11.02.2020
									03.03.2020
		Maewang	18.6306	98.84	*Allium cepa* L.				06.03.2020
**SOUTH AMERICA**	**Argentina**	Bahía Blanca	-39.3667	-62.6333	*Allium cepa* L.	15.08.2019	*Allium cepa* L.	Alfalfa and weeds	18.12.2019
	**Uruguay**	Progreso	-34.6163	-56.2232	*Allium cepa* L.	20.08.2019	Maize	Potato and grasses	19.12.2019
		Salto Grande	-31.2722	-57.8908	*Allium cepa* L.	06.06.2019	*Sorghum × drummondii*	Tomato, strawberry and sweet potato	17.12.2019
**NORTH AMERICA**	**Mexico**	Tulancingo de Bravo	20.1036	-98.3755	*Allium cepa* L.				30.07.2020
	**USA**	Huron	36.2800	-120.0814	*Allium cepa* L.	07.01.2020			29.07.2020

### DNA extraction

Thirty-three individual thrips (adults or larvae) were randomly chosen from each sample. In one case (Konan 08.08.2019) only 16 adult thrips were collected. DNA was extracted from individual thrips using the DNeasy^®^ Blood & Tissue Kit (QIAGEN, Valencia, CA), according to the suppliers’ recommendations.

### Amplification of the COI fragment and sequencing

DNA from individual thrips was used as the template for PCR amplification, using the universal COI primer pair, MTD7.2F ATTAGGAGCHCCHGAYATAGCATT and MTD9.2R CAGGCAAGATTAAAATATAAACTTCTG [30]. Twelve μl PCR solution was prepared according to the manual of the Qiagen Kit (6 μl Multiplex PCR kit solution, 0.25 μl for each 10 nM primer MTD7.2F and MTD9.2R, 3,5 μl of thrips DNA and 2 μl ddH2O). The PCR amplification protocol used was 95°C for 15 min, then 35 cycles of 95°C for 1min, 50°C for 1 min, and 72°C for 1 min, followed by a final extension at 72°C for 10 min. The PCR products were examined on a 1.5% agarose gel and sequenced in forward and reverse directions using a 3500 ABI Sequencer (Applied Biosystems, USA). The forward and reverse sequences from each band were used to build the consensus sequence with the package SeqMan Pro of the DNASTAR LASERGENE 17 program.

### PCR reaction to determine the reproductive mode of *T*. *tabaci*

The *T*. *tabaci* reproductive mode-specific PCR reaction was performed using three PCR primers, one universal (TCOR-ATTGCGTAAATTATTCCTAAAAGTCCA) and two reproduction-mode-specific primers: 1) mtCOA-SSP TCOS-AACAGCTATTCTCCTTCTTTATCTC, amplifying a fragment of 261 nucleotides in arrhenotokous reproducing thrips, and, 2) mtCOT-SSP TCOC-GAACAGTATATCCACCTTTATCAACG, amplifying a fragment of 451 nucleotides in case of thelytokous reproduction [15]. In the case of heteroplasmy, both fragments amplify at the same time [27].

DNA from individual thrips of the different *T*. *tabaci* haplotypes was used as the template for PCR amplification. Twelve μl of PCR solution was prepared according to the manual of the Qiagen Kit (6 μl Multiplex PCR kit, 0.25 μl for each strain-specific 10 μM primer mtCOA-SSP TCOS and mtCOT-SSP TCOC, 0.5 μl of the 10 μM primer TCOR, 3.5 μl of thrips DNA and 1.5 μl ddH2O). The PCR amplification protocol used was 95°C for 15 min, 35 cycles of 98°C for 10 s, 60°C for 1 min, and 68°C for 1 min, followed by a final extension at 72°C for 10 min. The size of PCR products was determined by 1.5% agarose gel electrophoresis, with a 100 bp DNA Ladder (Thermo Fisher Scientific) as a reference, and stained with RedSafe^™^. All the reproductive mode-specific DNA fragments obtained were purified and sequenced as described above. All the consensus sequences from the different *T*. *tabaci* haplotypes were aligned using CLUSTALW [31] in MEGA 7 [32] and grouped by the reproductive mode according to the size of the fragment (thelytokous and arrehenotokous, 451, and 261 nucleotides, respectively).

### *Thrips tabaci* reproductive mode estimation and distribution worldwide

The molecular determination of the reproductive mode [15] is not conclusive in the case of heteroplasmy [27]. Furthermore, it was also not possible to determine the reproductive mode via a progeny test because of the destructive methodology used for DNA extraction. Therefore, the reproductive mode of heteroplasmic individuals was derived from their position in the phylogenetic tree. A phylogenetic analysis was performed, including all *T*. *tabaci* haplotypes collected in our research plus the ones reported in the NCBI database for which the reproductive mode was indicated in the passport data or published papers. One *F*. *occidentalis* haplotype from NCBI was used as an outgroup. The congruence between the reproductive mode derived from mode-specific primers and the position in the phylogenetic tree was confirmed for non-heteroplasmic individuals. The frequency of thelytokous and arrhenotokous *T*. *tabaci* per location was calculated. A Chi-square test was carried out to determine if the thelytokous and arrhenotokous *T*. *tabaci* were evenly distributed over locations.

### Data processing and analyses

#### Database generation, COI sequences alignment, and primer trimming

A COI gene database was generated containing all the consensus sequences obtained from the individual thrips sequenced. All consensus sequences were ordered and grouped in MEGA 7 by location or location-time for those locations that were sampled more than once (Konan, Pulpi, and Sansai) (Table 1). Finally, all consensus sequences were aligned using CLUSALW [31] in MEGA 7 [32] after they had been trimmed from forward and reverse primer sequences.

#### Thrips haplotypes identification, frequency, and phylogenetic analysis

Different haplotypes were identified with the Pop Art program [33] and blasted to the NCBI database to identify the species they belong to. To analyze the relationship among the different haplotypes identified, a Neighbor-Joining tree was constructed with the Mega Align Pro package of the DNASTAR program. All thrips haplotype sequences detected have been submitted to the NCBI database, under accession numbers OQ603129-OQ603255.

#### Location or location-time thrips haplotype constitution and genetic distances

To depict which haplotypes were present at the sampled locations or locations-time, a stacked bar chart was made with InfoStat [34] using the haplotypes frequencies per location or location-time as input.

Nei’s genetic diversity was calculated as the number of base substitutions averaged over all pairwise comparisons in the thrips COI gene fragment by location or location-time using MEGA 7 [32]. Within a sample, Nei’s genetic diversity represents the average evolutionary divergence across the thrips sequence pairs among the 33 thrips analyzed per location or location-time. For samples with several species, the genetic diversity per population was calculated only for *T*. *tabaci* using the COI sequence.

To calculate the Nei’s genetic distance between *T*. *tabaci* populations the 545 *T*. *tabaci* sequences were grouped by location or location-time in MEGA 7 and the number of base substitutions per site was averaged over all sequence pairs between samples [32]. The genetic diversity within samples as well as the genetic distance between them were analyzed using the Maximum Composite Likelihood model [35] implemented in MEGA 7.

### Correlation between genetic distances and geographic distances

The correlation between the genetic and geographic distances, of the *T*. *tabaci* populations at the different locations, was calculated using the matrix of Nei genetic distances and the matrix of geographic distances among locations. For locations with more than one sampling time, each sample was considered a population, and the geographic distance between them a zero. The matrix of Nei genetic distances for the *T*. *tabaci* populations was calculated in MEGA 7. The matrix of geographic distances, among the different sampling locations, was calculated using the Diva Gis program [36]. The correlation between the genetic and geographical distance was calculated through a Mantel test conducted in Genalex 6.5 [37]. The geographic distance matrix was used as X and the genetic distance matrix as Y, and 999 permutations were applied.

### Retrieving and processing *T*. *tabaci* COI sequences from NCBI

All COI sequences of *T*. *tabaci* available in the NCBI database on November 25, 2022 were retrieved (https://www.ncbi.nlm.nih.gov/nuccore/?term=thrips+tabaci+coi). This resulted in a total of 504 sequences. Sequences were trimmed to obtain a fragment between positions 341 and 775 of the mitochondrial COI gene, that coincides with the fragment of our study. Sequences that missed more than five nucleotides at the beginning or end of the sequence compared to our COI gene fragment, were discarded. The *T*. *tabaci* haplotypes obtained in the present study were named H followed by a number, or HU in the case a haplotype was unique in our dataset (represented by one individual thrips only). A Neighbor-Joining tree [38] was constructed in MEGA 7 [32] based on the analysis of 170 sequences of *T*. *tabaci* and one of *F*. *occcidentalis* (accession JQ082479.1 from the NCBI database) as an outgroup (S1 Table). In the NJ tree, only different sequences are presented, whereas the identical ones are reported in a separate table. In the analysis, the option “pairwise deletion of the missing data” was used, since some NCBI sequences had up to five missing nucleotides due to the incomplete coverage (S1 Table). The genetic distances were computed using the Maximum Composite Likelihood method [35] and units are presented as the number of base substitutions per site.

## Results

### Diversity of thrips species in onion and related *Allium* species

**Fig 1:**
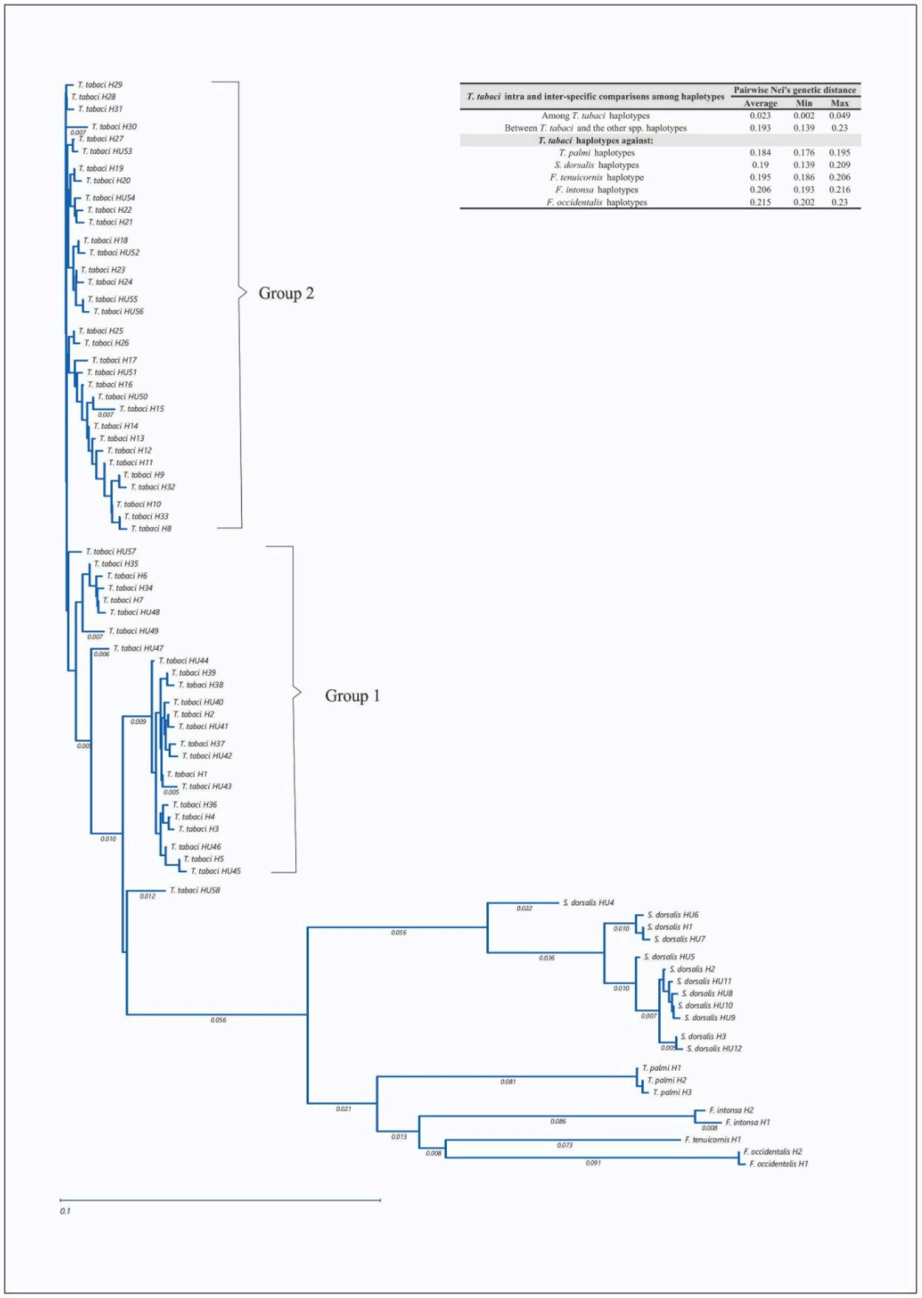
Neighbour-Joining tree of all 78 thrips COI haplotypes detected in the present study. The tree is based on 434 nucleotides of which 184 were polymorphic and 52 of them were polymorphic among *T*. *tabaci* haplotypes. Groups 1 and 2 contain *T*. *tabaci* haplotypes. The upper right chart shows the Pairwise Nei’s genetic distances among all *T*. *tabaci* haplotypes and between the *T*. *tabaci* haplotypes and *F*. *occidentales*, *F*. *tenuicornis*, *F*. *intonsa*, *T*. *palmi* and *S*. *dorsalis* haplotypes.

Five hundred and eighty-six individual thrips from 14 locations were sequenced for the COI region. All data can be found in S2 Table. From all thrips analyzed, 545 (93%) were *T*. *tabaci*. Other species found were *Scirtothrips dorsalis*, *Thrips palmi*, *Frankliniella intonsa*, *Frankliniella occidentalis* and *Frankliniella tenuicornis* (Fig 1). *Thrips tabaci* was present at all locations, while the other species were observed only at four of the 14 locations (Table 2). In Sansai (Thailand), four thrips species were found, *T*. *tabaci*, *S*. *dorsalis*, *T*. *palmi* and *F*. *intonsa*. In Pulpi (Spain) and Krabbendam (The Netherlands) two species were found, *T*. *tabaci* and *F*. *occidentalis*, whereas in De Kwakel (The Netherlands), one individual of *F*. *tenuicornis* was found next to *T*. *tabaci*. Thrips species different from *T*. *tabaci* were found in *Allium* spp. fields only, while in the onion fields all the thrips were *T*. *tabaci*.

**Table 2:** Thrips species detected at the 14 locations.

*Thrips* spp.	Frequency of *Thrips* spp.	Number of haplotypes	Location found
*Thrips tabaci*	9.30E-01	58	All
*Scirtothrips dorsalis*	4.27E-02	12	Sansai
*Thrips palmi*	1.37E-02	3	Sansai
*Frankliniella occidentalis*	6.80E-03	2	Pulpi, Krabbendam
*Frankliniella intonsa*	5.10E-03	2	Sansai
*Frankliniella tenuicornis*	1.70E-03	1	De Kwakel

**Fig 2:**
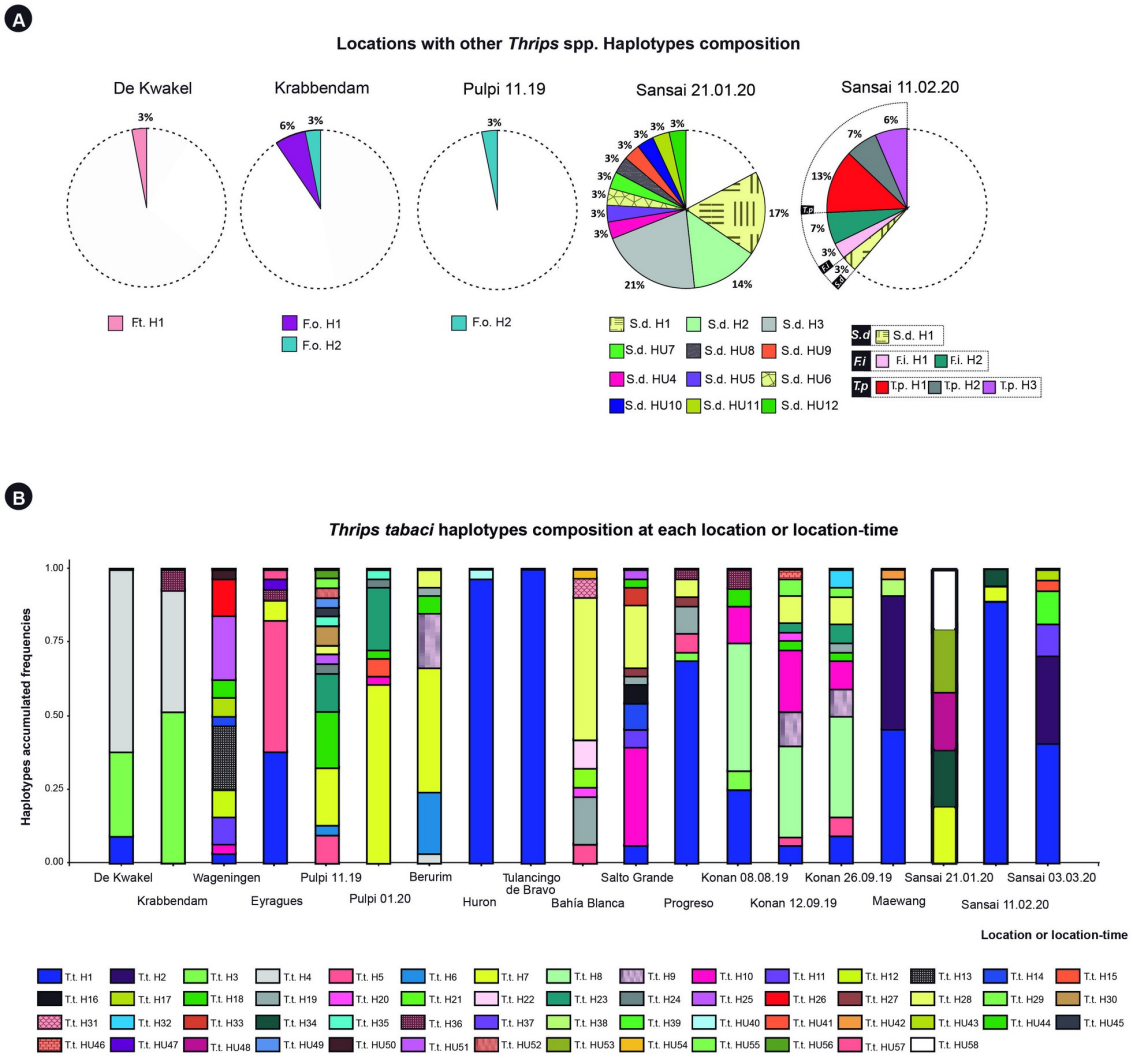
Composition of thrips haplotypes over collection sites and time points. **A)** Locations or location-time in which thrips haplotypes other than *T*. *tabaci* were found. The white parts of the circles correspond to *T*. *tabaci*. Other species and their haplotypes are indicated by different colors, see legend below the circles: F.t. *F*. *tenuicornis*, F.o. *F*. *occidentalis*, S.d. *S*. *dorsalis*, F.i. *F*. *intonsa*, T. p. *T*. *palmi*, **B)** *Thrips tabaci* haplotype composition at each location or location-time. The Y-axes show the frequencies of each haplotype present at the sampled location or location-time. Each haplotype is represented by a different color according to the color legend below the graph. Codes with an H represent the haplotypes with more than one individual thrips, and with HU the unique haplotypes.

The locations Pulpi and Sansai were sampled two and three times, respectively, and showed other species besides *T*. *tabaci* in the first or second sampling, while later in the season (last sampling) only *T*. *tabaci* was found (Fig 2; S3 Table). In Sansai, at the beginning of the season, the dominant species was *S*. *dorsalis* (85%) (Fig 2a, S3 Table). After 21 days, at the second sampling, the frequency of *S*. *dorsalis* had decreased to 3%, while *T*. *tabaci* had increased to 61%, and also *F*. *intonsa* (10%) and *T*. *palmi* (26%) were found. In the third sample, taken 20 days later, only *T*. *tabaci* was found (Fig 2b, S3 Table).

### Genetic diversity within *Thrips tabaci*

Among the 545 *T*. *tabaci* sequenced, 58 COI haplotypes were found (Table 3). The mean Nei’s genetic distance among *T*. *tabaci* haplotypes was 0.023. The mean genetic distance between the *T*. *tabaci* haplotypes and those of other species was 0.193 (Fig 1). All *T*. *tabaci* haplotypes were grouped in one clade consisting of two groups, while the haplotypes of the other species were grouped into species-specific clades (Fig 1).

**Table 3:** Variable sites in sthe 58 *T*. *tabaci* haplotype sequences and their frequencies.

***T*. *tabaci* haplotypes**	**Nucleotide positions**													**Frequency**	**Reproductive mode fragment length in base pairs**	**Phylogenetic group**
	4	7	9	10	14	16	37	46	54	79	85	88	99			
H1	T	A	T	A	A	T	C	G	T	A	A	G	T	0.284	451	**Group 1**
H2	.	.	.	.	T	.	.	.	.	.	.	.	.	0.042	451	
H3	.	.	.	.	.	.	.	.	.	.	.	.	.	0.048	451	
H4	.	.	.	.	.	.	.	.	.	.	.	A	.	0.061	451	
H5	.	.	.	.	.	.	.	.	.	G	.	.	.	0.042	451	
H6	.	.	.	.	.	.	G	.	.	.	.	A	.	0.015	451	
H7	.	.	.	.	.	.	G	.	.	.	.	A	.	0.081	451	
H34	.	.	.	.	T	.	G	.	.	.	.	A	.	0.004	451	
H35	.	.	.	.	.	.	G	.	.	.	.	A	.	0.004	451	
H36	.	.	.	.	.	.	.	.	.	.	.	.	.	0.009	451	
H37	.	.	.	T	T	.	.	.	.	.	.	.	.	0.006	451	
H38	.	.	.	.	.	.	.	.	.	.	.	.	.	0.004	451	
H39	.	.	.	.	.	.	.	.	.	.	.	.	.	0.006	451	
HU40	.	.	.	.	C	.	.	.	.	.	.	.	.	0.002	451	
HU41	.	.	.	.	T	.	.	.	.	.	.	.	.	0.002	451	
HU42	.	.	C	T	.	.	.	.	.	.	.	.	.	0.002	451	
HU43	.	.	.	.	.	.	.	.	.	.	.	.	.	0.002	451	
HU44	.	.	.	.	.	.	.	.	.	.	.	.	.	0.002	261 & 451	
HU45	.	.	.	.	.	.	.	.	.	G	.	.	.	0.002	451	
HU46	.	.	.	.	.	.	.	.	.	.	.	A	.	0.002	451	
HU47	.	.	.	.	.	.	.	.	.	.	.	A	.	0.002	261 & 451	
HU48	G	.	.	.	.	.	G	.	.	.	.	A	.	0.002	261 & 451	
HU49	.	.	.	.	.	.	.	.	.	.	.	A	.	0.002	451	
H8	.	.	.	.	.	.	.	.	.	.	.	A	.	0.051	261 & 451	**Group 2**
H9	.	.	.	.	.	.	.	.	.	.	G	A	.	0.024	261	
H10	.	.	.	.	.	.	.	.	.	.	.	A	.	0.046	261 & 451	
H11	.	.	.	.	.	.	.	.	.	.	.	A	.	0.009	261 & 451	
H12	.	.	.	.	.	.	.	A	.	.	.	A	.	0.006	261 & 451	
H13	.	.	.	.	.	.	.	A	.	.	.	A	.	0.013	261 & 451	
H14	.	.	.	.	.	.	.	.	.	.	.	A	.	0.007	261	
H15	.	.	.	.	.	.	.	.	.	.	.	A	.	0.004	261	
H16	.	.	.	.	.	.	.	.	.	.	.	A	.	0.004	261 & 451	
H17	.	.	.	.	.	.	.	A	.	.	.	A	.	0.004	261 & 451	
H18	.	.	.	.	.	.	.	.	.	.	.	A	.	0.026	261	
H19	.	.	.	.	.	.	.	.	.	.	.	A	.	0.020	261 & 451	
H20	.	.	.	.	.	.	.	.	.	.	.	A	.	0.004	261 & 451	
H21	.	.	.	.	.	.	.	.	.	.	.	A	A	0.004	261 & 451	
H22	.	.	.	.	.	.	.	.	.	.	.	A	A	0.006	261 & 451	
H23	.	.	.	.	.	.	.	.	.	.	.	A	.	0.026	261	
H24	.	.	.	.	.	.	.	.	.	.	.	A	.	0.004	261	
H25	.	.	.	.	.	.	.	A	.	.	.	A	.	0.015	261 & 451	
H26	.	.	.	.	T	.	.	A	.	.	.	A	.	0.007	261 & 451	
H27	.	.	.	.	T	.	.	.	.	.	.	A	.	0.004	261 & 451	
H28	.	.	.	.	.	.	.	.	.	.	.	A	.	0.061	261 & 451	
H29	.	.	.	T	.	.	.	.	.	.	.	A	.	0.006	261 & 451	
H30	.	G	.	.	.	.	.	.	.	.	.	A	.	0.004	261 & 451	
H31	.	.	.	.	.	.	.	.	.	.	.	A	.	0.004	261 & 451	
H32	.	.	.	.	.	.	.	.	.	.	G	A	.	0.004	261 & 451	
H33	.	.	.	.	.	.	.	.	.	.	.	A	.	0.004	261 & 451	
HU50	.	.	.	.	.	.	.	.	.	.	.	A	.	0.002	261	
HU51	.	.	.	.	.	.	.	.	.	.	.	A	.	0.002	261 & 451	
HU52	.	.	.	.	.	.	.	.	.	.	.	A	.	0.002	461	
HU53	.	.	.	.	T	.	.	.	.	.	.	A	.	0.002	261	
HU54	.	.	.	.	.	.	.	.	.	.	.	A	A	0.002	261 & 451	
HU55	.	.	.	.	.	.	.	.	G	.	.	A	.	0.002	261	
HU56	.	.	.	.	.	.	.	.	G	.	.	A	.	0.002	261	
HU57	.	.	.	.	.	C	.	.	.	.	.	A	.	0.002	261	
HU58	.	.	.	.	.	.	A	.	.	.	.	A	.	0.002		
***T*. *tabaci* haplotypes**	**Nucleotide positions**													**Frequency**	**Reproductive mode fragment length in base pairs**	**Phylogenetic group**
	100	111	115	118	121	131	148	169	175	190	193	197	200			
H1	A	T	A	G	T	G	A	T	G	A	T	T	A	0.284	451	**Group 1**
H2	.	.	.	.	.	.	.	.	.	.	.	.	.	0.042	451	
H3	.	.	.	.	.	.	.	.	.	.	.	.	.	0.048	451	
H4	.	.	.	.	.	.	.	.	.	.	.	.	.	0.061	451	
H5	.	.	.	.	.	.	.	.	.	.	.	.	.	0.042	451	
H6	.	.	.	A	.	.	.	A	.	G	.	.	.	0.015	451	
H7	.	.	.	A	.	.	.	.	.	G	.	.	.	0.081	451	
H34	.	.	.	A	.	.	.	.	.	G	.	.	.	0.004	451	
H35	.	.	.	A	.	.	.	.	.	G	.	.	.	0.004	451	
H36	.	.	.	.	.	.	.	.	.	.	.	.	.	0.009	451	
H37	.	.	.	.	.	.	.	.	.	.	.	.	.	0.006	451	
H38	.	.	.	.	.	.	.	A	.	.	.	.	.	0.004	451	
H39	.	.	.	.	.	.	.	A	.	.	.	.	.	0.006	451	
HU40	.	.	.	.	.	.	.	.	.	.	.	.	.	0.002	451	
HU41	.	.	.	.	.	.	.	A	.	.	.	.	.	0.002	451	
HU42	.	.	.	.	.	.	.	.	.	.	.	.	.	0.002	451	
HU43	.	.	.	.	.	.	.	.	.	.	.	.	.	0.002	451	
HU44	.	.	.	.	.	.	.	.	.	.	.	.	.	0.002	261 & 451	
HU45	.	.	.	.	.	A	.	.	.	.	.	.	.	0.002	451	
HU46	.	.	.	.	.	.	.	.	.	.	.	.	.	0.002	451	
HU47	G	.	.	A	.	.	.	.	.	G	.	.	.	0.002	261 & 451	
HU48	.	.	.	A	.	.	.	.	.	G	.	.	.	0.002	261 & 451	
HU49	.	.	.	A	.	.	G	.	.	G	.	.	.	0.002	451	
H8	G	.	.	A	.	.	.	.	A	G	.	.	.	0.051	261 & 451	**Group 2**
H9	G	.	.	A	.	.	.	.	A	G	.	.	.	0.024	261	
H10	G	.	.	A	.	.	.	.	A	G	.	.	.	0.046	261 & 451	
H11	G	.	.	A	.	.	.	.	A	G	.	.	.	0.009	261 & 451	
H12	G	.	.	A	.	.	.	.	A	G	.	.	.	0.006	261 & 451	
H13	G	.	.	A	.	.	.	.	.	G	.	.	.	0.013	261 & 451	
H14	G	.	.	A	.	.	.	.	.	G	.	.	.	0.007	261	
H15	G	.	G	A	.	.	.	.	.	G	.	.	G	0.004	261	
H16	G	.	.	A	.	.	.	.	.	G	.	.	.	0.004	261 & 451	
H17	G	.	.	A	.	.	.	.	.	G	.	.	.	0.004	261 & 451	
H18	G	.	G	A	.	.	.	A	.	G	.	.	.	0.026	261	
H19	G	.	.	A	.	.	.	A	.	G	.	.	.	0.020	261 & 451	
H20	G	.	.	A	A	.	.	A	.	G	.	.	.	0.004	261 & 451	
H21	G	C	.	A	.	.	.	.	.	G	.	.	.	0.004	261 & 451	
H22	G	.	.	A	A	.	.	.	.	G	.	.	.	0.006	261 & 451	
H23	G	.	G	A	.	.	.	.	.	G	.	.	.	0.026	261	
H24	G	.	G	A	.	.	.	.	.	G	.	.	.	0.004	261	
H25	G	.	.	A	.	.	.	.	.	G	.	.	.	0.015	261 & 451	
H26	G	.	.	A	.	.	.	.	.	G	.	.	.	0.007	261 & 451	
H27	G	.	.	A	.	.	.	.	.	G	.	.	.	0.004	261 & 451	
H28	G	.	.	A	.	.	.	.	.	G	.	.	.	0.061	261 & 451	
H29	G	.	.	A	.	.	.	.	.	G	.	.	.	0.006	261 & 451	
H30	G	.	.	A	.	.	.	.	.	G	.	.	.	0.004	261 & 451	
H31	G	.	.	A	.	.	.	.	.	G	.	.	.	0.004	261 & 451	
H32	G	.	.	A	.	.	C	.	A	G	.	.	.	0.004	261 & 451	
H33	G	.	.	A	.	.	.	.	A	G	.	.	.	0.004	261 & 451	
HU50	G	.	G	A	.	.	.	.	.	G	.	.	.	0.002	261	
HU51	G	.	.	A	.	.	.	.	.	G	.	.	.	0.002	261 & 451	
HU52	G	.	G	A	.	.	.	A	.	G	.	.	.	0.002	461	
HU53	G	.	.	A	.	.	.	A	.	G	.	.	.	0.002	261	
HU54	G	.	.	A	.	.	.	A	.	G	.	.	.	0.002	261 & 451	
HU55	G	.	G	A	.	.	.	.	.	G	.	.	.	0.002	261	
HU56	G	.	G	A	.	.	.	.	.	G	.	.	.	0.002	261	
HU57	G	.	.	A	.	.	.	.	.	G	.	.	.	0.002	261	
HU58	.	.	.	A	.	.	.	.	.	.	A	C	.	0.002		
***T*. *tabaci* haplotypes**	**Nucleotide positions**													**Frequency**	**Reproductive mode fragment length in base pairs**	**Phylogenetic group**
	208	209	245	247	268	277	287	289	292	295	308	310	322			
H1	T	A	G	A	C	T	G	C	T	T	T	G	G	0.284	451	**Group 1**
H2	.	.	.	.	.	.	.	.	.	.	.	.	.	0.042	451	
H3	.	.	.	G	.	.	.	.	.	.	.	.	.	0.048	451	
H4	.	.	.	G	.	.	.	.	.	.	.	.	.	0.061	451	
H5	.	.	.	.	.	.	.	.	.	.	.	A	.	0.042	451	
H6	.	.	.	.	.	.	.	T	.	C	C	.	A	0.015	451	
H7	.	.	.	.	.	.	.	T	.	C	C	.	A	0.081	451	
H34	.	.	.	.	.	.	.	T	.	C	C	.	A	0.004	451	
H35	.	.	.	.	.	.	.	T	.	C	C	.	A	0.004	451	
H36	.	.	.	.	.	.	.	.	.	.	.	.	.	0.009	451	
H37	.	.	.	.	.	.	.	.	.	.	.	.	.	0.006	451	
H38	.	.	.	.	.	.	.	.	.	.	.	.	.	0.004	451	
H39	.	.	.	.	.	.	.	.	.	.	.	.	.	0.006	451	
HU40	.	.	.	.	.	.	.	.	.	.	.	.	.	0.002	451	
HU41	.	.	.	.	.	.	.	.	.	.	.	.	.	0.002	451	
HU42	.	.	.	.	.	.	.	.	.	.	.	.	.	0.002	451	
HU43	.	.	.	.	.	.	A	.	.	.	.	.	.	0.002	451	
HU44	.	.	.	.	.	.	.	.	.	.	.	.	.	0.002	261 & 451	
HU45	.	.	.	.	.	.	.	.	.	.	.	A	.	0.002	451	
HU46	.	.	.	.	.	.	.	.	.	.	.	A	.	0.002	451	
HU47	.	.	.	.	.	.	.	.	.	C	C	A	.	0.002	261 & 451	
HU48	.	.	.	.	.	.	.	T	.	C	C	.	A	0.002	261 & 451	
HU49	C	.	.	.	.	.	.	T	.	C	C	.	A	0.002	451	
H8	.	T	C	.	.	.	.	T	.	C	C	A	A	0.051	261 & 451	**Group 2**
H9	.	.	.	.	.	.	.	T	.	C	C	A	A	0.024	261	
H10	.	.	.	.	.	.	.	T	.	C	C	A	A	0.046	261 & 451	
H11	.	.	.	.	.	.	.	T	.	C	C	.	A	0.009	261 & 451	
H12	.	.	.	.	.	.	.	T	.	C	C	.	A	0.006	261 & 451	
H13	.	.	.	.	.	.	.	T	.	C	C	.	A	0.013	261 & 451	
H14	.	.	.	.	.	.	.	T	.	C	C	.	A	0.007	261	
H15	.	.	.	.	.	.	.	G	A	C	C	.	A	0.004	261	
H16	.	.	.	.	.	.	.	T	.	C	C	.	A	0.004	261 & 451	
H17	.	.	.	.	.	.	.	T	.	C	C	.	A	0.004	261 & 451	
H18	.	.	.	.	.	.	.	T	.	C	C	.	A	0.026	261	
H19	.	.	.	.	.	.	.	T	.	C	C	.	A	0.020	261 & 451	
H20	.	.	.	.	.	.	.	T	.	C	C	.	A	0.004	261 & 451	
H21	.	.	.	.	.	.	.	T	.	C	C	.	A	0.004	261 & 451	
H22	.	.	.	.	.	.	.	T	.	C	C	.	A	0.006	261 & 451	
H23	.	.	.	.	.	.	.	T	.	C	C	.	A	0.026	261	
H24	.	.	.	.	.	.	.	T	.	C	C	.	A	0.004	261	
H25	.	.	.	.	.	.	.	T	.	C	C	.	A	0.015	261 & 451	
H26	.	.	.	.	.	.	.	T	.	C	C	.	A	0.007	261 & 451	
H27	.	.	.	.	.	.	.	T	.	C	C	.	A	0.004	261 & 451	
H28	.	.	.	.	.	.	.	T	.	C	C	.	A	0.061	261 & 451	
H29	.	.	.	.	.	.	.	T	.	C	C	.	A	0.006	261 & 451	
H30	.	.	.	.	G	.	.	T	.	C	C	.	A	0.004	261 & 451	
H31	.	.	.	.	.	G	.	T	.	C	C	.	A	0.004	261 & 451	
H32	.	.	.	.	.	.	.	T	.	C	C	A	A	0.004	261 & 451	
H33	.	T	.	.	.	.	.	T	.	C	C	A	A	0.004	261 & 451	
HU50	.	.	.	.	.	.	.	T	.	C	C	.	A	0.002	261	
HU51	.	.	.	.	.	.	.	T	.	C	C	.	A	0.002	261 & 451	
HU52	.	.	.	.	.	.	.	T	.	C	C	.	A	0.002	461	
HU53	.	.	.	.	.	.	.	T	.	C	C	.	A	0.002	261	
HU54	.	.	.	.	.	.	.	T	.	C	C	.	A	0.002	261 & 451	
HU55	.	.	.	.	.	.	.	T	.	C	C	.	A	0.002	261	
HU56	.	.	.	.	.	.	.	T	.	C	C	.	A	0.002	261	
HU57	.	.	.	.	.	.	.	T	.	C	C	A	A	0.002	261	
HU58	.	.	.	.	.	.	.	A	.	.	.	.	.	0.002		
***T*. *tabaci* haplotypes**	**Nucleotide positions**													**Frequency**	**Reproductive mode fragment length in base pairs**	**Phylogenetic group**
	331	355	361	364	382	385	388	391	400	406	417	418	429			
H1	C	C	T	C	A	G	A	G	T	T	T	T	G	0.284	451	**Group 1**
H2	.	.	.	.	.	.	.	.	.	.	.	.	.	0.042	451	
H3	.	.	.	.	.	A	.	.	.	.	.	.	.	0.048	451	
H4	.	.	.	.	.	A	.	.	.	.	.	.	.	0.061	451	
H5	.	.	.	.	.	A	.	.	.	.	.	.	.	0.042	451	
H6	.	T	.	T	.	.	.	T	C	.	.	.	.	0.015	451	
H7	.	T	.	T	.	.	.	T	C	.	.	.	.	0.081	451	
H34	.	T	.	T	.	.	.	T	C	.	.	.	.	0.004	451	
H35	.	T	.	T	.	.	.	A	C	.	.	.	.	0.004	451	
H36	.	.	.	.	.	A	.	.	.	.	.	.	.	0.009	451	
H37	.	.	.	.	.	.	.	.	.	.	.	.	.	0.006	451	
H38	.	.	.	.	.	.	.	.	.	.	A	.	.	0.004	451	
H39	.	.	.	.	.	.	.	.	.	.	.	.	.	0.006	451	
HU40	.	.	.	.	.	.	.	.	.	.	.	.	.	0.002	451	
HU41	.	.	.	.	.	.	.	.	.	.	.	.	.	0.002	451	
HU42	.	.	.	.	.	.	.	.	.	.	.	.	.	0.002	451	
HU43	.	.	.	.	.	.	.	.	.	.	.	.	A	0.002	451	
HU44	.	.	.	.	.	.	.	A	.	.	.	.	.	0.002	261 & 451	
HU45	.	.	.	.	.	A	.	.	.	.	.	.	.	0.002	451	
HU46	.	.	.	.	.	A	.	.	.	.	.	.	.	0.002	451	
HU47	.	.	.	T	.	A	.	A	C	.	.	.	.	0.002	261 & 451	
HU48	.	T	.	T	.	.	.	T	C	.	.	.	.	0.002	261 & 451	
HU49	T	T	.	T	.	.	.	A	C	.	.	.	.	0.002	451	
H8	.	.	C	T	G	.	.	A	C	C	.	C	.	0.051	261 & 451	**Group 2**
H9	.	.	C	T	G	.	.	A	C	C	.	C	.	0.024	261	
H10	.	.	C	T	G	.	.	A	C	C	.	C	.	0.046	261 & 451	
H11	.	.	C	T	G	.	.	A	C	C	.	C	.	0.009	261 & 451	
H12	.	.	C	T	G	.	.	A	C	.	.	C	.	0.006	261 & 451	
H13	.	.	C	T	G	.	.	A	C	.	.	C	.	0.013	261 & 451	
H14	.	.	C	T	G	.	.	A	C	.	.	C	.	0.007	261	
H15	.	.	C	T	G	.	.	A	C	.	.	C	.	0.004	261	
H16	.	T	C	T	G	.	.	A	C	.	.	C	.	0.004	261 & 451	
H17	.	.	.	T	G	.	.	A	C	.	.	C	.	0.004	261 & 451	
H18	.	T	.	T	G	.	.	A	C	.	.	.	.	0.026	261	
H19	.	T	.	T	G	.	.	A	C	.	.	.	.	0.020	261 & 451	
H20	.	T	.	T	G	.	.	A	C	.	.	.	.	0.004	261 & 451	
H21	.	T	.	T	G	.	.	A	C	.	.	.	.	0.004	261 & 451	
H22	.	T	.	T	G	.	.	A	C	.	.	.	.	0.006	261 & 451	
H23	.	T	.	T	G	.	.	A	C	.	.	.	.	0.026	261	
H24	.	T	.	T	G	.	.	A	C	C	.	.	.	0.004	261	
H25	.	T	.	T	G	.	.	A	C	.	.	.	.	0.015	261 & 451	
H26	.	T	.	T	G	.	.	A	C	.	.	.	.	0.007	261 & 451	
H27	.	T	.	T	G	.	.	A	C	.	.	.	.	0.004	261 & 451	
H28	.	T	.	T	G	.	.	A	C	.	.	.	.	0.061	261 & 451	
H29	.	T	.	T	G	.	.	A	C	.	.	.	.	0.006	261 & 451	
H30	.	T	.	T	G	.	G	A	C	.	.	.	.	0.004	261 & 451	
H31	.	T	.	T	G	.	.	A	C	.	.	.	.	0.004	261 & 451	
H32	.	.	C	T	G	.	.	A	C	C	.	C	.	0.004	261 & 451	
H33	.	.	C	T	G	.	.	A	C	C	.	C	.	0.004	261 & 451	
HU50	.	.	C	T	G	.	.	A	C	.	.	C	.	0.002	261	
HU51	.	.	C	T	G	.	.	A	C	.	.	.	.	0.002	261 & 451	
HU52	.	T	.	T	.	.	.	A	C	.	.	.	.	0.002	461	
HU53	.	T	.	T	G	.	.	A	C	.	.	.	.	0.002	261	
HU54	.	T	.	T	G	.	.	A	C	.	.	.	.	0.002	261 & 451	
HU55	.	T	.	T	G	.	.	A	C	.	.	.	.	0.002	261	
HU56	.	.	.	T	G	.	.	A	C	.	.	.	.	0.002	261	
HU57	.	T	.	T	G	.	.	A	C	.	.	.	.	0.002	261	
HU58	.	T	.	T	.	.	.	.	.	.	.	.	.	0.002		

Variable nucleotide positions in the 434 base pairs of the COI gene in the 58 *T*. *tabaci* haplotypes. The last three columns in the table show: 1) the frequency of each haplotype within the total set of sampled *T*. *tabaci*; 2) the fragments obtained using reproductive mode specific primers (461bp represent thelytoky, 261bp represent arrhenotoky or both); and 3) the two major *T*. *tabaci* phylogenetic groups of the NJ analysis represented by a green and orange bar. The haplotype HU58 does not belong to any group, nor showed bands for the reproductive mode-specific primers.

The most common haplotype of *T*. *tabaci* was H1 (28.4%), it was present at 10 of the 14 locations. In Tulancingo de Bravo, haplotype H1 was the only haplotype found. In Huron, H1 was the most important with a genetic diversity close to zero (Table 4, Fig 2b, S3 Table). The second most common haplotype was H7 (8%), present at 4 of the 14 locations (Eyragues, Pulpi, Berurim, and Sansai) followed by H4 and H28, both at a frequency of 6%. H4 was only found at the two locations in The Netherlands and in Berurim, while H28 was found in Asia, South America, and Europe (Fig 2b, S3 Table). H8 is another haplotype among the most common ones (5.1%). Haplotype H8 was only present in Konan at all three sampling times, as the most common haplotype. In addition, 19 unique (represented by only one thrips) haplotypes (HU40 to HU58) were identified considering all locations and time points together, representing 3.5% of all *T*. *tabaci* (Table 3). The largest genetic diversity among *T*. *tabaci* was observed in Konan, where 15 haplotypes were identified over three sampling times (Table 4).

**Table 4:** Nei’s genetic diversity of *T*. *tabaci* within locations or locations-time.

Location or location*time	Genetic diversity
De Kwakel	1.93E-03
Krabbendam	1.47E-03
Wageningen	9.04E-03
Eyragues	1.07E-02
Pulpi 11.19	1.62E-02
Pulpi 01.20	8.35E-03
Berurim	1.24E-02
Huron	1.40E-04
Tulancingo de Bravo	0.00E+00
Bahia Blanca	7.49E-03
Salto Grande	1.08E-02
Progreso	1.13E-02
Konan 08.08.19	2.51E-02
Konan 12.09.19	1.66E-02
Konan 26.09.19	1.92E-02
Maewang	2.02E-03
Sansai 21.01.20	1.72E-02
Sansai 11.02.20	5.95E-03
Sansai 03.03.20	2.62E-03

The dataset consisted of COI sequences of 434 nt from 545 *T*. *tabaci* collected at 14 locations. In Pulpi, Konan and Sansai, thrips were sampled respectively two, three and three times during the season, indicated by their sampling date.

### Changes in *T*. *tabaci* genetic diversity within locations over time

The three locations sampled more than once, Pulpi, Sansai, and Konan, showed changes in the genetic composition of *T*. *tabaci* over time, with the least diversity at the end of the season (Table 4, Fig 2b).

In Pulpi, 15 *T*. *tabaci* haplotypes were identified in the first sample. The three most frequent haplotypes were H7, H18, and H23 (Fig 2b, S3 Table). The second sample consisted of seven *T*. *tabaci* haplotypes of which H10 and H15 were new. Haplotypes H7 and H23 were the dominant haplotypes and their frequencies increased three and nearly two times, respectively. This resulted in a reduction in diversity, from 0.016 to 0.008 between samplings (Table 4).

In Sansai the first sample contained only five *T*. *tabaci* individuals (15%), all with a different haplotype (Fig 2b, S3 Table). In the second sample, the frequency of *T*. *tabaci* increased to 61%, of which 90% were the haplotype H1. The third sample contained *T*. *tabaci* only, again with H1 as the dominant haplotype (Fig 2b, S3 Table). Compared to the second sample, haplotypes H7 and H34 were no longer present, but five new haplotypes were observed (Fig 2b, S3 Table).

In Konan, all three samples consisted of *T*. *tabaci* only. In the first sample, seven haplotypes were identified with H8 as the dominant one (44%) followed by H1 (25%) (Fig 2b). In the second sample, 11 haplotypes were found, with H8 again as the most common (31%), followed by haplotypes H10 (21%), and H9 (12%). In the third sample, also 11 haplotypes were identified, with H19 and H32 as new haplotypes replacing H20 and HU46. H8 remained the most common haplotype (35%), followed by H9 (10%). The frequencies of other haplotypes were below 10% (Fig 2b, S3 Table).

### Genetic diversity within *Thrips tabaci* and mode of reproduction

Within the COI gene fragment of 434 nucleotides, 52 sites were polymorphic (Table 3). Based on the NJ tree (Fig 1), the 58 *T*. *tabaci* haplotypes clustered into two main groups, except for one haplotype (HU58) which did not belong to any group. Of the 58 haplotypes, 20 had the thelytokous fragment (451 bp) and were present in group 1 of the NJ tree, 11 haplotypes had the arrhenotokous fragment (261 bp) and were present in group 2, and 26 haplotypes had both fragments. Haplotype HU58 did not amplify in the reproductive mode PCR-specific reaction. Among the haplotypes having both fragments, three belonged to group 1, and 23 to group 2 (Fig 1 and S2 Fig).

Alignment of the sequences obtained for the 26 haplotypes with double (arrhenotokous and thelytokous) bands [15] showed that these fragments overlap. The arrhenotokous fragment overlapped from position 192 to the end of the thelytokous fragment. Within the thelytokous fragment sequence, there is a T insertion at the position 212 (S4 Table). This confirms the presence of heteroplasmy in haplotypes with both fragments since the arrhenotokous specific primer sequence is not present within the thelytokous fragment sequence.

The majority (72%) of individual *T*. *tabaci* analyzed belonged to nine common and widely spread haplotypes (H1 through H5, H7, H8, H10, H28) (Table 3, S3 Table), of which six haplotypes belonged to group 1 (Fig 1) and had the thelytokous band (Table 3), while the others (H8, H10, and H28) belonged to group 2 (Fig 1) with both bands and are thus heteroplasmic (Table 3). The other 28% of individual *T*. *tabaci* analyzed belonged to the 49 fewer common haplotypes with frequencies below 0.04. Seventeen of these haplotypes belonged to Group 1 and had either the thelytokous band (14) or both bands (3), while 31 belonged to Group 2 of which 11 had only the arrhenotokous band and 20 had both bands.

### *Thrips tabaci* geographic and genetic distances and distribution of reproductive mode

**Fig 3:**
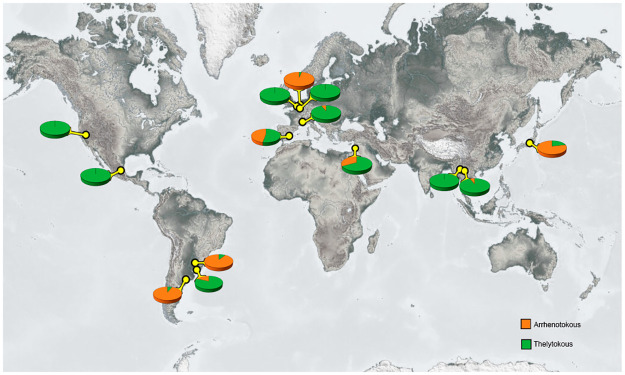
Distribution of arrhenotokous and thelytokous *T*. *tabaci* genotypes on onion and other *Allium* spp. Locations, where thrips were collected, are indicated by yellow dots. The reproductive mode is shown by pie charts.

No correlation was found between the matrixes of Nei’s genetic distances for the *T*. *tabaci* populations collected by us and the geographic distances between the locations where they were collected (R^2^: 5E-07; p-value: 0.428) (S1 Fig). The frequency of the *T*. *tabaci* reproductive mode per location is presented in Fig 3. The Chi-square test (*Χ*^2^) indicated significant differences between the observed thelytokous and arrhenotokous *T*. *tabaci* frequencies. The null hypothesis, assuming that both reproductive modes are evenly distributed per location around the world was rejected (*Χ*^2^ (13, *N* = 544) = 333.05, *p* = 2.83 E-63).

### Relationship between our *T*. *tabaci* haplotypes and NCBI accessions

A total of 112 accessions from the NCBI were aligned with our 58 *T*. *tabaci* haplotypes (S1 Table). The 112 NCBI accessions resulted in 45 different haplotypes because some of the NCBI accessions were identical for the 434 nucleotides COI gene fragment used in our study. Of these 45 haplotypes, 38 differed from the 58 haplotypes found in our study, resulting in a total of 96 different *T*. *tabaci* haplotypes. The phylogenetic tree, built with these *T*. *tabaci* haplotypes and *F*. *occidentalis* as an outgroup showed three main groups within *T*. *tabaci* (S2 Fig).

Group 1 consists of 49 haplotypes of which we collected 23 and 26 were reported in the NCBI database (S2 Fig). Among the haplotypes in Group 1, five of our haplotypes were reported earlier (see Table 5). An example is *T*. *tabaci* H1, which was collected in The Netherlands, France, USA, Mexico, Uruguay, Japan and Thailand, which is identical to 26 accessions from Pakistan collected in 2010 and 2011 and six accessions from China collected in 2011, 2018 and 2019. Group 2 in the phylogenetic tree consists of 41 haplotypes, representing 53 sequences (19 from the NCBI database plus 34 sequences from our study) (S2 Fig). Thirty-two of our haplotypes were not reported in the NCBI database (Table 5). The third Group defined in the phylogenetic tree is composed exclusively of five *T*. *tabaci* accessions from the NCBI collected in Greece in 2004 from tobacco plants.

**Table 5:** Haplotypes detected in our study already reported in the NCBI database and NCBI identical accessions considering the amplified 434 bp COI fragment.

Haplotypes or Accessions	Identical accessions numbers	Country and year of collection
**[T._tabaci_H28] (A)**	FN546159.1, FN546160.1, FN546161.1, FN546162.1, FN546163.1, FN546164.1, **AY196843.1 (A)**	[Spain, Israel, Argentina, Uruguay, Japan 2019–2020], United Kingdom 2009 (6), Greece 2004
**[T._tabaci_H23] (A)**	**AY196841.1 (A), AY196840.1 (A)**	[Spain, Japan 2019–2020], Greece 2004 (2)
KP845584.1 Pakistan 2011	KP845683.1, KP845808.1, KP845846.1	Pakistan 2011 (4)
AY196838.1 Greece 2004	AY196839.1	Greece 2004
**[T._tabaci_HU46] (T)**	KP845850.1, FN546156.1, **AB277236.1 (T)**, **MN036456.1 (T)**, LC325504.1, LC650835.1	[Japan 2019], Pakistan 2012, Bosnia-Herzegovina 2009, Japan 2004, China 2019, Japan 2016, Japan 2014
AM932012.1 Bosnia-Herzegovina 2004	**MN036455.1 (T)**, MH922170.1, MH922172.1	Bosnia-Herzegovina 2004, China 2018–2019 (3)
**[T._tabaci_H5] (T)**	**AY196835.1 (T)**, KP845636.1, LC650834.1, **MN036454.1 (T)**	[France, Spain, Argentina, Uruguay, Japan 2019–2020], Greece and Bulgaria 2004, Pakistan 2010, Japan 2014, China 2019
**[T._tabaci_H1] (T)**	KP845516.1, KP845523.1, KP845572.1, KP845578.1, KP845597.1, KP845600.1, KP845608.1, KP845611.1, KP845615.1, KP845618.1, KP845626.1, KP845634.1, KP845637.1, KP845656.1, KP845660.1, KP845661.1, KP845666.1, KP845694.1, KP845734.1, KP845755.1, KP845772.1, KP845773.1, KP845785.1, KP845807.1, KP845834.1, KP845861.1, **MN036453.1 (T)**, MN036458.1, MN036459.1, MN036460.1, MH922173.1, JF719579.1	[The Netherlands, France, USA, Mexico, Uruguay, Japan, Thailand 2019–2020], Pakistan 2010–2011 (26), China 2011-2018-2019 (6)
**[T._tabaci_H36] (T)**	**AB277237.1 (T)**, MH922171.1, FN546158.1	[The Netherlands, France, Uruguay, Japan 2019–2020], Japan 1996, China 2018, United Kingdom 2009
AM932007.1 Bosnia-Herzegovina 2004	AM932014.1	Bosnia-Herzegovina 2004
**[T._tabaci_H3] (T)**	**AY196831.1 (T)**, FN546157.1, **MN036457.1 (T)**, **AB277235.1 (T)**, AM931998.1, AM932043.1, FN546151.1, FN546154.1, FN546165.1, FN546167.1, FN546168.1	[The Netherlands, Uruguay, Japan 2019], Switzerland 2002, Bosnia-Herzegovina 2009, China 2019, Japan 2001, United Kingdom 2004-2005-2009 (7)

Data that belong to the present study is shown between rectangular brackets. The number of accessions from the same country is shown between brackets after the country. Accessions or haplotypes in bold are reported as arrhenotokous **(A)** or thelytokous **(T)**, based on our study, NCBI passport data, or papers [13, 14, 24] (S1 Table).

## Discussion

### *Thrips tabaci* is the main species in onion cultivation and *Allium* spp, but not the only one

This research aimed to study thrips diversity in fields with onion or its related species around the world. We set out a comprehensive study in which we sequenced 33 randomly chosen individual thrips per location or location-time, allowing us to identify with a 95% certainty, all genotypes (haplotypes) present in the population with a frequency of at least 0.1 [39]. Some locations were sampled more than once during the season, making it possible to analyze changes in the thrips population over time. Thrips species identification was based on the homology between the COI gene sequences and the data reported in the NCBI database. The range of genetic distance found in the 434 bp COI gene fragment within species did not overlap with the range of genetic distance found between species meaning that the amplified COI region of the gene is a good fragment to distinguish *T*. *tabaci* from other species. The genetic distance between *T*. *tabaci* haplotypes was in all cases below 5%, which is similar to other studies that used the same COI primers set. Furthermore, the average genetic distance between *T*. *tabaci* haplotypes and the other species’ haplotypes was around 20%, also in agreement with previous studies [26, 30, 40]. So, the 434 bp COI gene fragment used in our study showed good discriminative power between species and, at the same time detected ample genetic variation within species, strengthening its suitability to detect genetic variation between and within species and populations.

We found that the main species of thrips that affects onion and its related species is *Thrips tabaci*, which is in agreement with previous studies [5, 29, 41, 42]. Other thrips species such as *Thrips palmi*, *Scirtothrips dorsalis*, *Frankliniella tenuicornis*, *Frankliniella intonsa*, and *Frankliniella occidentalis*, were present at specific locations. The presence of *F*. *occidentalis* has previously been reported in onions in Tanzania [43] and Georgia [28]. In Georgia, the tobacco thrips, *Frankliniella fusca*, was the most important species in onion [28]. However, the number of incidents with *T*. *tabaci* in onions in Georgia is increasing [28], which might be explained by the fact that *T*. *tabaci* outcompetes *F*. *fusca* on onion [44]. The occurrence of *F*. *intonsa* in *A*. *cepa* and *A*. *fistulosum* has also been reported in Poland [29]. *Thrips palmi* and *S*. *dorsalis* are commonly reported in melon and pepper, respectively [45] and both thrips species occur occasionally in *Allium spp*. [46, 47]. Finally, in our study, we report for the first time *F*. *tenuicornis* in *Allium*. Until now, *F*. *tenuicornis* was reported as specific for wild and cultivated Gramineae [48].

Thrips species other than *T*. *tabaci* were found at certain locations only. Which thrips species are found is likely to be determined by the climatical conditions and centers of origin of the species. Sansai is located in the tropics, which may explain the presence of thrips species like *S*. *dorsalis* and *T*. *palmi* which are typical for these regions [49, 50]. In general, thrips species diversity is larger in the tropics and neotropics than in temperate regions [51]. The presence of pepper as a former crop in Sansai may explain the presence of *S*. *dorsalis*, commonly known as chili thrips. Pepper is the main host of *S*. *dorsalis* [52], but its host range includes a wide diversity of plant taxa from different families that enable *S*. *dorsalis* to remain in an area during intercropping [53]. The presence of *F*. *intonsa* at Sansai might be associated with the presence of *Allium fistulosum* in the *Allium* spp field since it has been reported as the common host plant for *F*. *intonsa* [29]. Apart from that, the *Allium* field in Sansai was surrounded by several (crop) species, including banana, bamboo, and herbs such as marigold and weeds, from which thrips may have moved into the field. In the case of De Kwakel, the field was surrounded by wild Gramineae grasses which are the typical host of *F*. *tenuicornis* [48], which might explain its presence. As a consequence, a crop should be considered as a unit integrated with the adjacent and previous vegetation [54, 55] and not as an isolated island.

Our results also show that the change in species composition at certain locations can be fast and dynamic, with *T*. *tabaci* as the unique species present at the end of the season. Phenomena that may be explained by a combination of factors, including a higher competition capacity [44], higher reproductive rate [56], and migration of the other species [57].

In the sampled onion fields only *T*. *tabaci* was found. In fields planted with *Allium* spp., other thrips species were found as well, although not all *Allium* spp. fields sampled had thrips species other than *T*. *tabaci*. Whether the more diverse set of host plants in the *Allium* spp. fields has played a role in shaping the thrips communities and populations remains to be established. Our setup does not allow us to draw any firm conclusions on this.

### *Thrips tabaci* diversity

#### Groups and reproductive systemc

The *T*. *tabaci* haplotypes obtained from onion and related species clustered into two phylogenetic groups. The haplotypes within group 1 (Fig 1) may be classified as thelytokous since all of them grouped in the same cluster with the haplotypes previously reported as thelytokous in the NCBI (S2 Fig, Table 5 and S1 Table) and they showed the PCR fragment which is specific for the thelytokous mode of reproduction (Table 3). The haplotypes within group 2 may be classified as arrhenotokous since they grouped with the arrhenotokous haplotypes reported in the NCBI and they amplified the PCR fragment specific for the arrhenotokous mode of reproduction. However, both groups also contained haplotypes that amplified both specific fragments, a phenomenon known as heteroplasmy [27]. Heteroplasmy was more common within group 2 (68% against 13% in group 1). As a consequence, the PCR reactions [15] were not conclusive in determining the reproductive mode. We assume that using the phylogenetic position (group 1 or 2) may be the best way to determine the reproductive mode in these cases, but more progeny tests are needed to conclusively establish that all heteroplasmic individuals in a certain group indeed display the expected mode of reproduction.

We found all the heteroplasmic haplotypes (except HU48) in populations that contained individuals of both phylogenetic groups (sympatric distribution), suggesting that they are the result of crossings between members of these groups. Such crossings have already been reported [20, 21]. The high percentage of heteroplasmy in group 2 (arrhenotokous) might be explained by the frequent paternal leakage [58] during sexual reproduction among arrhenotokous haplotypes, while within group 1 there is no occurrence of that since the haplotypes reproduce parthenogenetically. In both groups heteroplasmy in successive generations would be maintained via the mother line [27], if selection and genetic drift do not act against it [59].

Novel variation seems to be generated. We found clear examples of mutations. One individual at Huron (haplotype HU40) is only one base pair different from H1, and therefore a likely descendant of that haplotype. Also, recombination between different COI haplotypes that coexist in heteroplasmic individuals may be a source of novel variants, a mechanism likely to occur in group 2, where the percentage of heteroplasmy is high. We found 51 *T*. *tabaci* haplotypes not reported before in the NCBI database of which the majority (32) belongs to the group 2 (arrhenotokous lineage).

The low frequency of most arrhenotokous haplotypes is in agreement with their limited presence in the NCBI database. Many arrhenotokous haplotypes might have been undetected in former studies due to limited sampling efforts [39]. Since we sampled quite extensively, more arrhenotokous haplotypes present in low frequency were detected. Another explanation may be the narrow host range of arrhenotokous thrips compared to thelytokous [16]. The absence or limited presence of suitable hosts for arrhenotokous thrips during certain periods of the year may lead to bottlenecks in their populations reducing the haplotype frequencies.

In our study, six out of the nine more frequent haplotypes were thelytokous and showed a global distribution, which might be expected from their generalist nature [16]. Of the less frequent 49 haplotypes, 31 belong to group 2. These haplotypes may represent the less adapted or recently emerged haplotypes, as most of them are restricted to a specific location and are arrhenotokous.

*Thrips tabaci* is reported in more than 120 countries, but only for 29 countries, there is molecular data related to reproductive mode. Thelytokous thrips were found in all 29 countries while the arrhenotokous were reported in 16 countries only [16]. We found arrhenotokous *T*. *tabaci* for the first time in Argentina, Uruguay, France, Spain, and Thailand, and we confirmed their presence in Israel [17], Japan [13], and The Netherlands [17].

Thelytokous and arrhenotokous *T*. *tabaci* were not evenly distributed around the world. The thelytokous thrips (group 1) were present at all sampled locations (Fig 3), and five populations were composed solely of thelytokous haplotypes. Only a few arrhenotokous haplotypes showed a relatively high frequency in certain populations. The uneven distribution of both reproductive modes around the world might be explained by the availability of arrhenotokous preferred hosts [16] throughout locations and years.

#### *Thrips tabaci*, population diversity and dynamics over time

Based on our results the diversity of *T*. *tabaci* within locations is reduced towards the end of the season, suggesting that some genotypes (haplotypes) have higher fitness compared to others. For example, H8 in Konan (Japan), H1 in Sansai (Thailand), and H7 in Pulpi (Spain) were the haplotypes most frequent across the season at these locations. In the case of H8 a positive interaction between haplotype and location might be expected since this haplotype is exclusively present in Konan. For the haplotypes H1 and H7 an overall higher fitness might be inferred since they were found at several locations. It will be interesting to see if the most frequent haplotypes across the season at certain locations are maintained across different years.

Different levels of genetic diversity are reported for *T*. *tabaci* populations from different locations or years [24, 41, 43, 60]. Based on our findings it is clear that the diversity of *T*. *tabaci* populations not only varies within a season but also among locations. The absence or nearly zero genetic diversity in Tulancingo de Bravo (Mexico) and Huron (USA) is not commonly reported and might point to a relatively recent invasion of *T*. *tabaci* or a poor adaptation of other haplotypes. On the other hand, highly diverse *T*. *tabaci* populations were also detected, distant from each other and composed of several haplotypes from different phylogenetic groups.

Genetic diversity in pests is expected to be high in the regions that coincide with the center of origin of the pest, and lower in places that were invaded recently [49]. High diversity at locations far away from the presumed center of origin points at invasions that took place a long time ago, resulting in well-adapted haplotypes from both phylogenetic groups. We found the most diverse *T*. *tabaci* populations both close to the center of origin [3] (Pulpi) as well as far away in Konan, Progreso, and Salto Grande (Table 4, Fig 2b). This result may also indicate that the center of origin of *T*. *tabaci* is larger than originally thought [3], or that some locations could be considered as secondary centers of diversification, former introduction sites in which adaptation and generation of new diversity took place.

The distribution pattern of the haplotypes throughout the world and their frequencies at different locations explain the lack of correlation between the geographical and genetic distances. This lack of correlation has also been reported in a study within China with *T*. *tabaci* populations [24] and *F*. *occidentalis* populations [61]. The fact that distant populations are genetically similar might be explained by the spread of the haplotypes by people [61]. Plant trading and agriculture have already been suggested as key activities in the spread of other insect pests such as *Bemisia tabaci* [62]. Human activities could result in high gene flow and low genetic variability between locations at a large scale [24]. The finding that nine *T*. *tabaci* haplotypes (H1, H28, H5, H19, H10, H3, H36, H23, and H27) are present both in Uruguay (Salto Grande, North, and Progreso, South) and Konan (Japan) can most probably be explained as the result of commercial trade of garlic and/or onion as a cause of the gene flow between these distant locations.

## Conclusions

*Thrips tabaci* was the main species present in *Allium* fields. Nevertheless, other thrips species were also found in small numbers. Thrips other than *T*. *tabaci* were found at specific locations with diverse surroundings, mainly in the tropics and particularly frequent at the beginning of the growing season. In *T*. *tabaci* the genetic distance among haplotypes was below 5% and two different phylogenetic groups were found, which can be linked to the reproductive modes thelytokous (group 1) and arrhenotokous (group 2). Within group 2 there was a remarkable presence of heteroplasmic individuals. The thelytokous thrips were more frequent and worldwide distributed, while arrhenotokous thrips have a more restricted distribution and were always found in sympatry with the thelytokous thrips. The level of genetic diversity within populations differed among locations. Some *T*. *tabaci* haplotypes were present in a high number at different locations worldwide. No correlation was found between the genetic and geographic distances, suggesting that human activities may have spread thrips haplotypes throughout the world.

## Supporting information

S1 Fig Geographical versus genetic distance of the sampled thrips populations. Nei’s genetic distances between the *T*. *tabaci* populations sampled at 14 different locations and the geographic distances in kilometres between the locations. There was no correlation between the Nei’s genetic distances among *T*. *tabaci* populations at the different locations and its geographical distances (Mantel test p value: 0.428).
(TIF)

S2 Fig Neighbor-Joining tree showing the relationships between the 58 *T*. *tabaci* haplotypes of the present study (represented as H or HU and the number) and 38 other *T*. *tabaci* haplotypes previously reported in the NCBI database (represented by accession number) and 1 sequence from NCBI database of *F*. *occidentalis* as an outgroup. The COI gene fragment used consisted of 434 nucleotide positions. Branch lengths is based on the number of base substitutions per site. The average genetic distance among all *T*. *tabaci* sequences in the phylogenetic tree was 0.027. Next to the accession or haplotype name, the number of times a specific fragment was found among the 112 NCBI accessions numbers and our 58 haplotypes is shown between brackets, and further detailed in Table 5. Group 1 is regarded as thelytokous thrips based on the passport data from the NCBI database or former papers [13, 14, 24], accessions reported as thelytokous are colored in green, accessions or haplotypes identical to accessions reported with thelytokous reproductive system are within a green rectangle (Table 5 and S1 Table). Group 2 is regarded as arrhenotokous thrips, the accessions with this reproductive mode are colored in orange, accessions or haplotypes identical to accessions reported as arrhenotokous are within an orange rectangle (Table 5 and S1 Table). Group 3 represents the tobacco group [14].
(TIF)

S1 Table Passport data and sequences of the 112 *T tabaci* and 1 *F*. *occidentalis* NCBI accessions used to build the neighbor joining tree together with the sequences of the 58 haplotypes detected in our study. In the last two columns the total sites and coverage of the NCBI accessions are depicted in comparison with the haplotypes found in our study after all sequences aligned and trimmed before and after position 1 and 434 respectively of the fragment amplified in our study.
(XLSX)

S2 Table Sequences of the 434 bp fragment of the COI gene of 586 thrips sampled at 14 locations worldwide. For each individual thrips the haplotype and species are presented. The latter is based on a BLAST against the sequences in the NCBI database.
(XLSX)

S3 Table Thrips haplotypes frequencies per location or location time. a) Thrips haplotypes frequencies considering all the thrips sequenced per sample. b) *Thrips tabaci* haplotype frequencies per sample considering only the *T*. *tabaci* in the samples that were originally composed by multiple thrips species. In all cases the frequencies are colored by conditional formatting from red to white in descendent order respectively.
(XLSX)

S4 Table COI gene fragments generated in the reproductive mode specific PCR reaction for thrips that showed double bands. The different COI fragments are grouped per reproductive type.
(XLSX)
