# Supplementary figures and images for: Genetic diversity of thrips populations on Allium species around the world
Source: PLoS One. 2023 Aug 17;18(8):e0289984. doi: 10.1371/journal.pone.0289984 (PMC10434924; doi:10.1371/journal.pone.0289984)

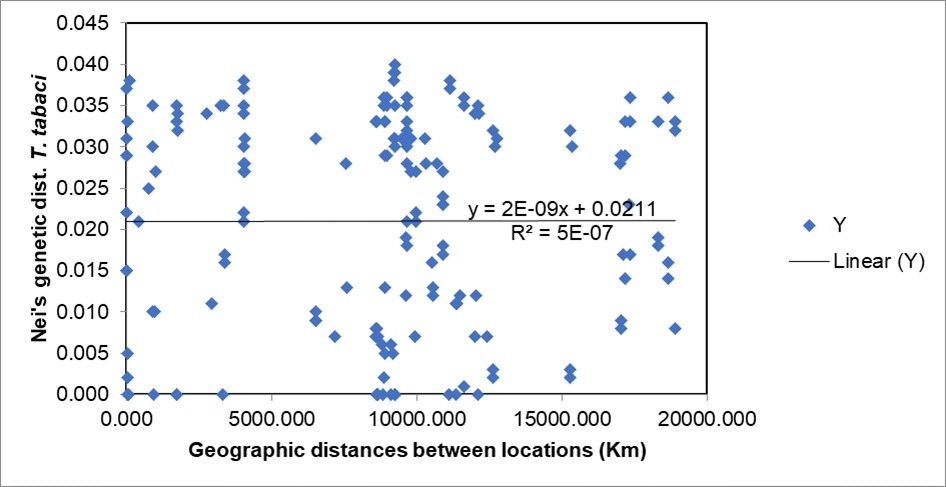

Supplement: S1 Fig — Nei’s genetic distances between the T. tabaci populations sampled at 14 different locations and the geographic distances in kilometres between the locations. There was no correlation between the Nei’s genetic distances among T. tabaci populations at the different locations and its geographical distances (Mantel test p value: 0.428). (TIF) [file pone.0289984.s001.tif]

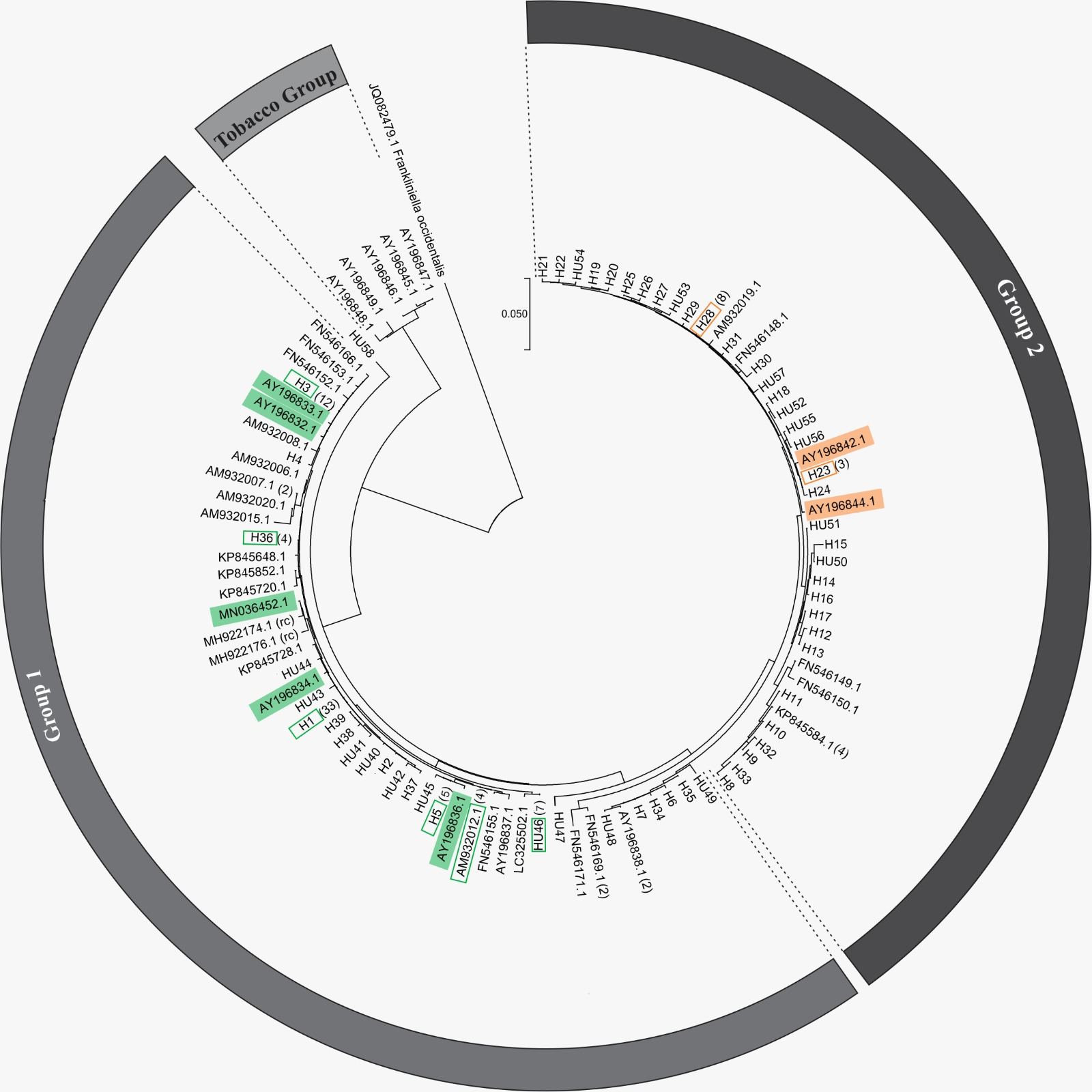

Supplement: S2 Fig — The COI gene fragment used consisted of 434 nucleotide positions. Branch lengths is based on the number of base substitutions per site. The average genetic distance among all T. tabaci sequences in the phylogenetic tree was 0.027. Next to the accession or haplotype name, the number of times a specific fragment was found among the 112 NCBI accessions numbers and our 58 haplotypes is shown between brackets, and further detailed in Table 5. Group 1 is regarded as thelytokous thrips based on the passport data from the NCBI database or former papers [13, 14, 24], accessions reported as thelytokous are colored in green, accessions or haplotypes identical to accessions reported with thelytokous reproductive system are within a green rectangle (Table 5 and S1 Table). Group 2 is regarded as arrhenotokous thrips, the accessions with this reproductive mode are colored in orange, accessions or haplotypes identical to accessions reported as arrhenotokous are within an orange rectangle (Table 5 and S1 Table). Group 3 represents the tobacco group [14]. (TIF) [file pone.0289984.s002.tif]
